# Mastitis and related management factors in certified organic dairy herds in Sweden

**DOI:** 10.1186/1751-0147-48-11

**Published:** 2006-07-17

**Authors:** Cecilia Hamilton, Ulf Emanuelson, Kristina Forslund, Ingrid Hansson, Torkel Ekman

**Affiliations:** 1 Division of Ruminant Medicine and Veterinary Epidemiology, Department of Clinical Sciences, P.O. Box 7054, Swedish University of Agricultural Sciences, SE-750 07 Uppsala, Sweden; 2 Department of Biomedical Sciences and Veterinary Public Health, P.O. Box 7009, Swedish University of Agricultural Sciences, SE-750 07 Uppsala, Sweden; 3 Swedish Dairy Association, Box 210, SE-101 24 Stockholm, Sweden

## Abstract

**Background:**

Mastitis is one of the major threats to animal health, in organic farming as well as conventional. Preliminary studies of organic dairy herds have indicated better udder health in such herds, as compared to conventional herds. The aim of this paper was to further study mastitis and management related factors in certified organic dairy herds.

**Methods:**

An observational study of 26 certified organic dairy herds in mid-eastern Sweden was conducted during one year. A large-animal practitioner visited the herds three times and clinically examined and sampled cows, and collected information about general health and management routines. Data on milk production and disorders treated by a veterinarian in the 26 herds, as well as in 1102 conventional herds, were retrieved from official records. Multivariable logistic regression was used to assess associations between herd type (organic vs. conventional) and incidence of disorders.

**Results:**

The organic herds that took part in the study ranged in size from 12 to 64 cows, in milk production from 3772 to 10334 kg per cow and year, and in bulk milk somatic cell counts from 83000 to 280000 cells/ml. The organic herds were found to have a lower incidence of clinical mastitis, teat injuries, and a lower proportion of cows with a high somatic cell count (as indicated by the UDS, Udder Disease Score) compared to conventional herds. The spectrum of udder pathogenic bacteria was similar to that found in other Swedish studies. Treatment of mastitis was found to be similar to what is practised in conventional herds. Homeopathic remedies were not widely used in the treatment of clinical mastitis.

The calves in most of these organic herds suckled their dams for only a few days, which were not considered to substantially affect the udder health. The main management factor that was different from conventional herds was the feeding strategy, where organic herds used a larger share of forage.

**Conclusion:**

Udder health in Swedish organic herds appears to be better than in conventional herds of comparable size and production. The major difference in management between the two types of farms is the proportion of concentrates fed. The mechanisms explaining the association between intensity of feeding and udder health in dairy cows require further research.

## Background

Mastitis is one of the major threats to animal health, in organic farming as well as conventional. Mastitis therapy accounts for a very large proportion of antibiotic drug use in dairy production [1-4] and one of the aims of organic production is to reduce the use of antibiotics [5]. Thus, according to the standards of organic production, animals treated with such restricted substances are subject to doubled withdrawal periods before milk may be sold to the dairy. To be able to reduce use of antibiotics it is important to keep the animals healthy by providing optimal care, feed and housing. Concern about the well-being of cows on organic dairy farms because of dietary restrictions has, however, been voiced among veterinarians. Our preliminary studies of organic dairy farms indicated better udder health in such herds, as compared to conventional herds [6,7], although no differences in overall animal health and welfare could be identified in other studies [8]. This paper will focus on aspects of udder health.

## Methods

### Participating farms

All family farms in four counties in mid-eastern Sweden, that had produced milk according to the organic standards for at least two full years, were asked to participate in the study. Of 37 eligible farms, 26 participated throughout the study, which was carried out during 12 months. In addition, all conventional herds of the same size (13 to 65 cows), located in the same region of Sweden and belonging to the same livestock association and the same dairy co-operative as the organic herds, were identified (n = 1102).

### Farm visits

An experienced large animal veterinarian visited the organic farms on three occasions, in November, March and July. The farms had in total 823 cows and at the first visit a third of the lactating cows in each herd was randomly selected (n = 257), except four cows that were to be culled before the next visit. Each selected cow was examined at each visit for general health, body condition and cleanness. Udder health was assessed using California Mastitis Test (CMT) at the first visit. Quarters with a severe case of mastitis, indicated by a CMT score ≥4, were sampled and samples sent to the mastitis laboratory at the National Veterinary Institute (SVA) in Uppsala for bacteriological culturing.

The farmers were interviewed on matters such as feeding and milking, and routines around calving.

### Health journal

Each farmer kept a journal for the purpose of noting all health-related events in the herd during the year, including method of treatment, where applicable. Culled cows and reasons for culling were also entered in the journal.

The farmers were asked to record cases of mastitis, defined as a cow having clinical, local and/or general signs of udder disease in one or more quarters. Milk samples from cases of mastitis were taken with a device specially designed to minimize the risk for contamination and mix-up of udder quarters (Mastistrip^©^, SVA, Uppsala, Sweden; [9]) for analysis at SVA. A convenience sample of 27 cows with an udder disease score 4 and higher at drying off, were also sampled by the farmers with Mastistrip^©^. Udder disease score (UDS) is a measure of the udder health of an individual cow, based upon three consecutive months of test milking results of individual cow somatic cell counts [10,11]. Udder disease score are graded 0–9, where each figure indicates a 10% increase of the probability that the cow has infectious mastitis, i.e. a UDS 0–2 imply a probability of infection of 0–29%, and corresponds approximately to having a SCC of <131 000 cells/ml on three subsequent test milkings.

### Questionnaire

The farmers responded to a questionnaire regarding general farm data. It also contained questions on feeding, milking, and grooming routines, and other details concerning the herd.

### Official records

Milk production was measured at monthly production tests in 25 of the 26 herds, according to standard procedure, and registered by the official Swedish milk-recording scheme run by the Swedish Dairy Association. One herd did not participate in the milk-recording scheme. For that herd the milk yield was estimated on the basis of the amount of milk delivered to the dairy. For cows where the milk test was preceded by a nursing calf, the farmer and/or the authors estimated the amount of milk consumed by the calf by comparing the milk yield in a day the calf was not allowed to suckle with the milk yield when the calf had suckled, and the total amount was registered.

Data on yearly herd average milk production and bulk milk somatic cell counts (BMSCC), retrieved from the Swedish Dairy Association, and data on disorders treated by a veterinarian, retrieved from the national animal disease recording system (NADRS; [12]), were available for the 25 organic herds as above and for the 1102 conventional herds as above.

Data on pathological findings at slaughter were retrieved from the Swedish Board of Agriculture and from abattoirs as described by Hansson et al. [13].

### Statistical methods

Multivariable logistic regression, as applied in the SAS macro GLIMMIX [14], was used to assess the association between herd type (organic vs. conventional) and presence of disease, as recorded in the NADRS, average proportion of cows in UDS 0–2 and 6–9, and proportion of cows that had been in UDS 6–9 at least once during the study period. Presence of disease was measured as annual incidence densities (AID), calculated as (total number of cases/total number of cow-days in herd) × 365 × 100, thus representing the number of cases per 100 cow-years. Annual incidence densities were calculated for veterinary-treated cases of mastitis, teat injuries, and culling due to mastitis.

The possibly confounding variables average lactation number, herd size, and milk production were introduced to the logistic regression model as continuous variables. Breed was introduced as a categorical variable.

The model output is reported in terms of the logit function, i.e. the log-odds, but was transformed to probabilities using the inverse link to express the least square means on the original scale. Confidence intervals were also computed on the logit scale and converted by the inverse link function.

## Results

The mean herd size was 32 cows (range 13–64) for the organic herds and 33 cows (range 13–65) for the selected conventional herds. Mean milk yield per cow was 6213 kg (range 3772–10334) and 7572 kg (3802–11379) for the organic and conventional herds, respectively, while corresponding numbers for geometric mean BMSCC were 173000 cells/ml (range 83000–280000) and 191000 cells/ml (range 45000–540000).

### Housing and feeding

The most common type of housing was a tie-stall barn. This was found on 20 of the farms. Rubber stall mats were used on four farms, and six farms had rubber stall mats in some stalls. Ten farms had no stall mats. Bedding material was straw in varying amounts, in three cases mixed with sawdust or peat moss. Six farms had free-stalls, three equipped with cubicles, and three farms had loose housing systems with deep litter straw beds. Free stalls with cubicles had rubber stall mats, on two farms bedded with straw and one with sawdust.

The total grain and concentrate ration ranged from 7 kg to 12 kg per day. Four farmers fed only barley and oats. The others also used one or more of wheat, peas, beet pulp and molasses, soybean meal and canola meal, fed separately or as a commercial concentrate. Vitamin and mineral supplements were fed every day according to general recommendation on 18 farms, somewhat less than the recommended dose on five farms, "once in a while" on two farms and not at all on one farm.

The forage consisted of silage with a large proportion of legumes, mostly red and white clover, and hay. In three herds forage made up approximately 70% of total daily dry matter, and in remaining herds 50 to 60%.

### Suckling calves

In all herds the calves were allowed to suckle the dam during the first days after birth. This would take place either in the calving pen, in the free-stall, or the calf was free in the barn and the cow tied in her stall. In 10 herds the calves would suckle the dam for one to two weeks, and in two herds the calves were free in the barn, able to suckle the dam at will, throughout the milk period of 10 weeks. The strategy of letting calves suckle lactating cows with subclinical or clinical mastitis was used sparingly.

### Treatment of mastitis

From interview and questionnaire it could be seen that farmers' readiness to call a veterinarian when a cow was found to have mastitis varied. Only two farmers would call when there was only a slight change in the milk, and one farmer would not call the veterinarian for any regular case of mastitis. When there was only a slight change in the milk most farmers would apply massage with liniment and strip milk the affected quarter, and only call for a veterinarian if the cow showed systemic signs of disease.

Six farmers used homeopathic remedies to a varying extent but not exclusively. Homeopathic remedies were used more often to treat other ailments or trauma, such as butted udders and injured teats, than clinical mastitis. Cows had also quarters with mastitis blinded, and were later culled, as a means to improve udder health in the herd. Cows with blinded quarters were usually not bred again, but there were examples of cows having blinded quarters that still stayed in the herd for several lactations. The number of cows with blinded quarters in the herds ranged from 0 to 12%, with an average of 4%.

The number of cows culled due to mastitis was similar in organic and conventional herds, according to the official records (Table 1).

**Table 1:** Distribution of udder health related parameters in 25 of the organic herds in the study and 1102 conventional herds of the same size and in the same local dairy association. The means and 95% confidence intervals (CI) are derived from multivariable logistic regression analyses and have been corrected for the effects of milk yield, herd size, breed, and lactation number, while ranges are as observed

	Organic			Conventional			
			
	Mean	CI	Range	Mean	CI	Range	p-value^1^
Mastitis treatments, AID^2^	9	6–14	0–30	15	13–16	0–100	0.038
Teat injuries, AID	0.3	0–2	0–3	2	1–2	0–42	0.048
Annual average prevalence of cows in UDS^3 ^0–2	74	69–78	56–98	64	62–65	25–98	<0.001
Annual average prevalence of cows in UDS 6–9	10	8–13	0–20	15	14–15	0–49	0.002
Percentage of cows at least once during the year in UDS 6–9	23	18–28	0–50	32	31–36	0–84	<0.001
Culled due to mastitis	8	6–12	0–28	8	7–9	0–44	NS

^1 ^Statistical test of difference between organic and conventional herds as estimated in the multivariable logistic regression analyses.

^2 ^Annual incidence density.

^3 ^Udder disease scores (UDS) are graded 0–9, where each figure indicates a 10% increase of the probability that the cow has infectious mastitis

### Subclinical mastitis

According to the official records, organic farms had more cows in low UDS than the conventional farms, and fewer in higher UDS. The estimated proportion of cows that had been in UDS 6–9 at least once during the year was higher in conventional than in organic herds (Table 1). The differences were statistically significant (p < 0.01).

Sampling at the initial farm visit revealed 66 cows (26%) with a CMT score ≥4 in a total of 104 quarters. Pathogenic bacteria were found in 52% of the quarter samples. The most prevalent bacterium was *Staphylococcus aureus *(*S. aureus*) while Coagulase negative staphylococci (CNS) were the second most common bacteria (Table 2). Fifteen of the 18 cows with *S. aureus *had UDS 4 or higher and six were culled before drying off.

**Table 2:** Results of bacteriological cultures of milk samples of a) 104 quarters (66 cows) with a California Mastitis Test ≥ 4 at the initial udder health examination of 257 cows; b) 38 cases of clinical mastitis yielding 42 sampled quarters (one harboured two pathogens); and c) 107 udder quarters in 27 cows with UDS^1^ ≥ 4 at drying off (one cow had a blinded quarter)

	Initial examination		Clinical mastitis^2^		Drying off^2^	
Culture results	N	%	N	%	N	%

*Staphylococcus aureus *(of which β-lactamase prod.)	19 (2)	18.3 (10)	5 (1)	11.9 (20)	7 (0)	6.5 (0)
Coagulase negative staphylococci (of which β-lactamase prod.)	17 (5)	16.4 (30)	0	0.0	7 (3)	6.5 (43)
*Streptococcus uberis*	8	7.7	3	7.0	5	4.7
*Streptococcus dysgalactiae*	10	9.6	4	9.3	2	1.9
*Escherichia coli*			8	18.6		
*Arcanobacterium pyogenes*			1	2.3		
Mixed growth	10	9.6	1	2.4		
Negative	40	38.5	21	50.0	86	80.0

^1 ^Udder disease score (UDS) are graded 0–9, where each figure indicates a 10% increase of the probability that the cow has infectious mastitis.

^2 ^Samples taken by farmers using Mastistrip^®^.

### Clinical mastitis

The estimated incidence of clinical mastitis treated by a veterinarian as derived from NADRS was 9.1 per 100 cows in the organic herds, and 14.7 in the conventional herds (Table 1). This difference was statistically significant (p < 0.05).

In the farmers' health journals there were 96 cases of clinical mastitis recorded, ranging in signs from slight to severe, giving an incidence of 11.7%. Samples were taken with Mastistrip^© ^from all four quarters from 38 of these cases and cultured. Only quarters with clinical mastitis are presented here. Four of the cows were recorded as showing signs of mastitis in two quarters; thus results from cultures of 42 quarters are presented in Table 2. One quarter harboured two pathogens. Seven cows in seven herds were infected with *Escherichia coli *(*E. coli*), one cow in two quarters. Twenty-one quarter samples (50%) were negative and one sample showed growth of mixed culture.

Organic herds had a statistically significantly lower estimated incidence (p < 0.05) of teat injuries (Table 1). During the study period there were three organic herds that each had one case of injured teats treated by veterinarian, amounting to an incidence of 0.25, versus 1.76 among the conventional.

### Drying off-samples

Infection with pathogenic bacteria was detected in 13 cows (48%) and 21 udder quarters in the cows sampled at drying off. Results are presented in Table 2.

Of the 18 cows that had growth of *S. aureus *at the initial examination 8 were sampled again at drying off. Four still showed growth of *S. aureus*, one was infected with CNS, one had mixed growth and two were negative.

### Findings at slaughter

Four of 91 cows (4.4%) that were slaughtered during the study year were classified as having mastitis.

## Discussion

### Clinical mastitis

The incidence of clinical mastitis treated by a veterinarian and recorded in the NADRS was significantly lower in the organic herds than in the conventional. This could be the result of either a truly better udder health, or a lower treatment rate as registered in the NADRS.

The incidence of cases of clinical mastitis treated by a veterinarian was lower than the actual incidence evidenced by the health journals that the farmers kept during the study period. This was in agreement with the farmers' statements regarding when, in terms of the signs at hand, to call a veterinarian. Most farmers would apply massage and frequent milking to mild cases. Elander & Hallén-Sandgren [15] found that the farmers with a low rate of mastitis cases treated by a veterinarian used more alternative treatment methods, such as frequent hand stripping, culling, blinding of mastitic quarters, or using homeopathic remedies, than farmers with a high rate of cases.

Homeopathy was not a commonly used method of treatment for mastitis in this study. It has been assumed that organic farmers hesitate to call the veterinarian for medical treatment because of the costs of the doubled withdrawal time. Because of this they may be more inclined to use homeopathic remedies. The results from this and other studies [15,16] suggest that the readiness to call the veterinarian is similar among organic and conventional farmers in Sweden, with a wide range within both groups. Zwald et al. [17], however, found in the USA that conventional dairy producers were more likely to use advice from veterinarians for recommendations of treatment of mastitis while organic dairy producers were more likely to rely on advice from other farmers.

The spectrum of pathogenic bacteria found in the present study was similar to that found in other Swedish studies of udder pathogens in samples taken by veterinarians examining cases of clinical mastitis [18]. Infections with *E. coli *were numerically more common in the organic herds, whereas the incidence of infections with *S. aureus*, *Streptococcus uberis *and *Streptococcus dysgalactiae *were in the same order in the two studies.

The rate of culling due to mastitis was similar in the organic and conventional herds, in spite of the organic herds having a lower incidence of clinical mastitis. This may indicate that culling of cows with chronic mastitis was used as a strategy to improve udder health in the organic herds, which is in agreement with results from conventional Swedish dairy herds [16].

### Subclinical mastitis and dry cow therapy

The percentage of cows in high and low UDS indicates a better udder health in the organic herds than in the conventional. The average BMSCC also points in the same direction. However, the herd somatic cell count would be biased by the use of nurse cows if a large number of cows with poor udder health were to bypass the test milking by moving over to the calf pen. Only on a few occasions, however, did farmers speak of cows whose udder health had improved while suckled, and subsequently returned to milk production.

In a study of findings at slaughter comprising 1238 organically and 204744 conventionally managed cows in Sweden Hansson et al. [13] found that 7.8% of slaughtered conventional cows and 3.9% of organic cows had mastitis (p < 0.001). In the present study a similar incidence (4.4%) was found. This further indicates that udder health is better in organic than in conventional Swedish dairy herds.

### Management and mastitis

Housing conditions and management practices found in this study showed no major differences to conventional farms in other Swedish studies [15,16]. Also Sato et al. [19] concluded that little differed between the two farm types with respect to management parameters.

The main difference appears to be the amount of concentrate fed to the cows. Differences in feeding was also found in a study in the USA where total mixed rations and purchased feeds were used on more conventional dairy farms compared with organic dairy farms [17], and Sato et al. [19] found differences in grazing intensity between the two farm types. Although the association between ration and disease in dairy cattle has been studied extensively, only few studies have been able to demonstrate an effect on udder health by changing feeding regime or amount of concentrate. Barnouin et al. [20], in an epidemiological study, found that a high level of energy in relation to protein was a risk factor for clinical mastitis. Johnson and Otterby [21] found a weak (p < 0.10) association between a dry period ration containing 47% grain and higher CMT values as compared with a ration with 12% grain. Klug et al. [22] had statistically significantly more cases of clinical mastitis among cows and heifers fed a similar high grain ration during lactation. Ekman [16] found a statistically significant association between level of nutrition and a higher incidence of clinical mastitis and lower bulk tank somatic cell count. Some studies report numerical differences in mastitis incidence rates between different feeding regimes but due to too few cows no statistically significant results were obtained [23-25]. A recent Swedish study of risk factors associated with a high treatment incidence of clinical mastitis identified several feeding related risk factors (Nyman, unpublished results).

Although few, these reports support the hypothesis that the difference seen in udder health between organic and conventional farms may be due to the lower levels of grain and concentrates fed on organic farms. Lower levels of concentrate would mean a less stressed rumen metabolism [26]. The reason for a higher incidence of mastitis on farms with high concentrate levels may be increased milk yield and forced udder metabolism. More research is needed to further elucidate the mechanisms and the associations between high levels of concentrates and udder disease in dairy cows.

## Conclusion

Udder health in Swedish organic herds appears to be better than in conventional herds of comparable size and production. The major difference in management between the two types of farms is the proportion of concentrates fed. The mechanisms explaining the correlation between intensity of feeding and udder health in dairy cows require further research.

## Competing interests

The author(s) declare that they have no competing interests.

## Authors' contributions

CH carried out the field study, compiled the results and drafted the manuscript. UE participated in the design of the study, compiled and analysed the official data. KF initiated the study, participated in the design and coordination of the study. IH participated in the field study. TE initiated the study, participated in the design and coordination of the study. All authors read and approved the manuscript.
